# Peptidylarginine Deiminase (PAD) and Post-Translational Protein Deimination—Novel Insights into Alveolata Metabolism, Epigenetic Regulation and Host–Pathogen Interactions

**DOI:** 10.3390/biology10030177

**Published:** 2021-02-26

**Authors:** Árni Kristmundsson, Ásthildur Erlingsdóttir, Sigrun Lange

**Affiliations:** 1 Institute for Experimental Pathology at Keldur, University of Iceland, Keldnavegur 3, 112 Reykjavik, Iceland; asthildure@hi.is; 2 Tissue Architecture and Regeneration Research Group, School of Life Sciences, University of Westminster, London W1W 6UW, UK

**Keywords:** protein deimination/citrullination, peptidylarginine deiminase (PAD), Alveolata, Apicomplexa, Chromerida, gene-regulation, histone, metabolism, host–pathogen interaction, *Piridium sociabile*, *Merocystis kathae*

## Abstract

**Simple Summary:**

Alveolates are a major group of free living and parasitic organisms; some of which are serious pathogens of animals and humans. Apicomplexans and chromerids are two phyla belonging to the alveolates. Apicomplexans are obligate intracellular parasites; that cannot complete their life cycle without exploiting a suitable host. Chromerids are mostly photoautotrophs as they can obtain energy from sunlight; and are considered ancestors of the apicomplexans. The pathogenicity and life cycle strategies differ significantly between parasitic alveolates; with some causing major losses in host populations while others seem harmless to the host. As the life cycles of some are still poorly understood, a better understanding of the factors which can affect the parasitic alveolates’ life cycles and survival is of great importance and may aid in new biomarker discovery. This study assessed new mechanisms relating to changes in protein structure and function (so-called “deimination” or “citrullination”) in two key parasites—an apicomplexan and a chromerid—to assess the pathways affected by this protein modification. Our findings point to novel regulatory mechanisms in these parasites’ lifecycles via protein deimination and may provide novel insights into their adaptability to different environments and hosts as well as host–pathogen coevolution.

**Abstract:**

The alveolates (Superphylum Alveolata) comprise a group of primarily single-celled eukaryotes that have adopted extremely diverse modes of nutrition, such as predation, photoautotrophy and parasitism. The alveolates consists of several major phyla including the apicomplexans, a large group of unicellular, spore forming obligate intracellular parasites, and chromerids, which are believed to be the phototrophic ancestors of the parasitic apicomplexans. Molecular pathways involved in Alveolata host–pathogen interactions, epigenetic regulation and metabolism in parasite development remain to be fully understood. Peptidylarginine deiminases (PADs) are a phylogenetically conserved enzyme family which causes post-translational protein deimination, affecting protein function through the conversion of arginine to citrulline in a wide range of target proteins, contributing to protein moonlighting in physiological and pathological processes. The identification of deiminated protein targets in alveolate parasites may therefore provide novel insight into pathogen survival and host-pathogen interactions. The current study assessed PAD homologues and deiminated protein profiles of two alveolate parasites, *Piridium sociabile* (Chromerida) and *Merocystis kathae* (Apicomplexa). Histological analysis verified strong cytoplasmic PAD expression in both Alveolates, detected deiminated proteins in nuclear and cytoplasmic compartments of the alveolate parasites and verified the presence of citrullinated histone H3 in Alveolata nucleus, indicating roles in epigenetic regulation. Histone H3 citrullination was also found significantly elevated in the host tissue, indicative of neutrophil extracellular trap formation, a host-defence mechanism against a range of pathogens, particularly those that are too large for phagocytosis. Proteomic analysis of deiminated proteins from both Alveolata identified GO and KEGG pathways strongly relating to metabolic and genetic regulation, with some species-specific differences between the apicomplexan and the chromerid. Our findings provide novel insights into roles for the conserved PAD/ADI enzyme family in the regulation of metabolic and epigenetic pathways in alveolate parasites, possibly also relating to their life cycle and host–pathogen interactions.

## 1. Introduction

The alveolates (Superphylum Alveolata) comprise a group of primarily single-celled eukaryotes that have adopted extremely diverse modes of nutrition, such as predation, photoautotrophy and parasitism. Generally, the alveolates are split into three major phyla, Apicomplexa, Ciliophora and Dinoflagellata. However, three additional lineages, the colpodellids, chromerids and perkinsids, are also considered alveolates, but they do not fit within any of the three major phyla [1,2,3,4,5]. Phylum Apicomplexa consist of more than 6000 nominal species. These are unicellular and spore-forming pathogens, which share a defining feature, the apical complex that comprises a system of structural and secretory elements that facilitates interaction with the host cell. They are obligate parasites, which exhibit both asexual (merogony) and sexual (gamogony) reproduction followed by the development of infective sporozoites (sporogony), represented by numerous different life forms, either in a single hosts (termed monoxenous) or by an additional intermediate host (heteroxenous) [6]. In most cases they develop intracellularly, although epi- and extracellular development is also known [7]. The pathogenicity of apicomplexans varies considerably between species and/or their hosts. Due to their obligate intracellular location (with exceptions), they cause some pathology in all cases; some are considered to have low pathogenicity while others are highly pathogenic. Some species are serious human pathogens causing diseases such as malaria (*Plasmodium* spp.) and toxoplasmosis (*Toxoplasma gondii*), while others cause coccidiosis in various lower vertebrates and invertebrates [8], such as *Merocystis kathae*, which infects the renal organ of the common whelk (*Buccinum undatum*) and the muscles of the pectinid bivalves [9]. Until recently, the Phylum Chromerida consisted of two unicellular, photosynthetic coral-associated species, i.e., *Chromera velia* and *Vitrella brassicaformis* [2,10]. The discovery of *C. velia* [2], which has been termed “the mother of all parasites” [11], was a milestone with regard to understanding the evolution of parasitism; a vital component in protist research, concerning both parasites and free-living unicellular organisms. The discovery of *Vitrella brassicaformis* few years later provided further support of the evolutionary relationship of chromerids and apicomplexans [10]. In 2019, one additional species, *Piridium sociabile*, a parasite of the common whelk, was assigned to the Phylum Chromerida [12]. Prior to that, *P. sociabile* was considered an apicomplexan [13]. In terms of phylogeny, it is exceptional, being the only parasite within this phylum and a link between free living/symbiotic photosynthetic organisms and parasites [12]. To date, it is commonly acknowledged that parasitic apicomplexans have evolved from phototrophic ancestors, with the chromerids considered their closest relatives [12,14,15].

As discussed above, the two Alveolata species, *M. kathae* and *P. sociabile*, known to infect the common whelk, are quite different in terms of phylogeny [9,12,13,16,17]. *M. kathae* is a heteroxenous apicomplexan, with the common whelk as a definitive host and pectinid bivalves as intermediate hosts [9]. *M. kathae* has recently been reassigned to a new class, Marosporida *class nov*., along with a number of other apicomplexans infecting marine invertebrates [18]. *M. kathae* is considered harmless to its whelk host but has been shown to be highly pathogenic in pectinid bivalves, and is believed to be responsible for mass mortality events in Iceland [19] and a suspected cause for epidemics in the Northwest Atlantic and Alaska [20]. *P. sociabile* infects the foot of the common whelk, it does not seem to be pathogenic to its host and its life cycle is mostly unclear [9,13]. However, ongoing studies suggest it has a free-living flagellated life stage (Kristmundsson et al., unpublished data), similar to two other known chromerids, i.e., *C. velia* and *V. brassicaformis* [2,10].

Peptidylarginine deiminases (PADs) are a phylogenetically conserved calcium-dependent family of enzymes with multifaceted roles in health and disease. In mammals five PAD isozymes are known, while three PAD isozymes have been described in birds and reptiles, but only one PAD in teleost and cartilaginous fish [21,22,23,24,25,26]. Evidence for PADs has recently also been reported in Mollusca, Crustacea and Merostomata [27,28,29]. Furthermore, PAD homologues, also referred to as arginine deiminases (ADI) [30] have been described lower in phylogeny, including in parasites [31] and bacteria [32,33], as well as in fungi [34]. ADI have indeed been identified in some apicomplexans including *Babesia ovata* (XP_028866800.1) and *Gregarina niphandrodes* (XP_011128800.1), but no studies have hitherto been carried out assessing their roles in post-translationally mediated processes in Alveolata. PADs convert arginine into citrulline in an irreversible manner, leading to post-translational modification (citrullination/deimination) in numerous target proteins of cytoplasmic, nuclear and mitochondrial origin [21,23,24,35,36,37]. Deimination causes structural protein changes which can affect protein function and, consequently, downstream protein–protein interactions. Deimination can amongst other contribute to neo-epitope generation, which results in inflammatory responses, as well as affecting gene regulation via deimination of histones [38,39,40,41,42]. PADs are furthermore a key driver of neutrophil extracellular trap formation (NET/ETosis), a phylogenetically conserved antipathogenic mechanism against a number of parasitic, bacterial and viral pathogens [43,44,45,46,47], including alveolates parasitising pinnipeds and cetaceans [48]. In addition, pathogens have been found to use their own ADI to modify host immune proteins, facilitating pathogen immune evasion [32,49], as well as neutralising other competing pathogens [50]. As post-translational changes contribute to protein moonlighting, which allows one protein to exhibit different functions within one polypeptide chain [51], post-translational deimination may form part of a mechanism facilitating such functional diversity. Therefore, deimination-mediated regulation of homologous and conserved proteins in the phylogenetic tree may provide information on the diversification of the functions of immune and metabolic pathways throughout evolution and in host–pathogen interactions.

A majority of the studies on PADs and downstream deimination have hitherto related to human pathological mechanisms, but recently a comparative body of research has focused on identifying putative roles for PADs in physiological and immunological pathways in a wide range of taxa throughout the phylogenetic tree, including terrestrial and marine mammals, reptiles, birds, bony- and cartilaginous fish, Mollusca, Myrostomata and Crustacea [23,24,25,26,27,28,29,36,52,53,54,55]. PADs have indeed been identified to have roles in mucosal, innate and adaptive immunity in a range of taxa [23,24,25,26,27,28,29,36,52,53,54,56,57,58]. Importantly, PADs have also been identified as important players in infection and antipathogenic responses, including antiviral [59,60], antibacterial [32,33] and antiparasitic ones [31].

The current study assessed putative PAD homologues and post-translational protein deimination signatures in two parasitic alveolates, both of which infect the common whelk. This study provides novel insights into Apicomplexa and Chromerida’s epigenetic regulation, metabolism and host–pathogen interactions, adding to current understanding of the roles for post-translational modifications in the functional diversification of conserved proteins throughout phylogeny.

## 2. Materials and Methods

### 2.1. Alveolata Collection

Common whelks, *Buccinum undatum*, were collected in Breidafjördur, West Iceland (65°7.576′ N; 22°44.738′ W), using whelk traps. The two alveolates were collected from infected whelks according to previously described methods [9,12], with slight modifications. Whelks were sedated using 0.1% MgSO_4_ in seawater for 1–2 h, then examined for the presence of *P. sociabile* cysts on the surface of the foot and *M. kathae* in the kidney, using a dissecting microscope. Mature (large) cysts were gently squeezed with pointed forceps until the parasites were released from the infected tissues. The resulting exudate was collected into concave glass spot plates containing sterile PBS. To remove as much host tissue as possible, the samples were subsequently rinsed three times in PBS. For protein analysis, the rinsed samples, containing the protozoans were put in 1 mL Eppendorf tubes and frozen at −80 °C until further use for the individual experiments.

For histological analysis, common whelk kidney tissue infected with *M. kathae*, or foot muscle containing *P. sociabile* were fixed in Davidson’s fixative, and prepared according to conventional histological methods, i.e., embedded in paraffin and tissue sections cut at 5 µm.

### 2.2. Histology

Paraffin tissue sections were deparaffinised in xylene, followed by sequential rehydration from 100, 90, 70% alcohol and taken to water. Immunohistochemistry was carried out according to previously established protocols [23,24]. In brief, demasking was performed by heating (11 min in the microwave at power 6) in citric acid buffer (pH 6.0), blocking was in 5% goat serum and primary antibodies were applied over night at 4 °C as follows: pancitrulline antibody (F95, MABN328, Merck, Feltham, UK; diluted 1/100), PAD2 antibody (ab50257, Abcam, Cambridge, UK; diluted 1/100) and antihistone H3 citrullination antibody (ab5103, Abcam; diluted 1/100). Washing was with 100 mM phosphate buffer, secondary antibody incubation with antirabbit IgG or antimouse IgM biotinylated antibodies (Vector laboratories, Peterborough, UK; diluted 1/200) for 1 h at RT, followed by Avidin-Biotinylated peroxidase Complex (ABC, Vector Laboratories) incubation for 1 h and colour development was carried out using diaminobenzidine/hydrogen peroxide (DAB) stain. Background staining was carried out using Mayer’s haematoxylin (Sigma, Gillingham, UK).

### 2.3. Protein Extraction

Protein was extracted using RIPA+ buffer (Sigma) containing protease inhibitor cocktail (P8340, Sigma) by homogenising the protozoans by pipetting gently up and down 20 times and then extracting protein for 2 h on ice with gentle shaking and regular pipetting. The suspension was centrifuged at 16,000× *g* for 20 min and the supernatant containing the proteins was collected. The protein extracts were further assessed by SDS-PAGE followed by silver staining or Western blotting, and by proteomic analysis (LC-MS/MS) following F95 enrichment as described in Section 2.4, Section 2.5, Section 2.6 and Section 2.7.

### 2.4. Isolation of Deiminated Proteins—F95 Enrichment

Total deiminated proteins were isolated from the protein extracts of the two Alveolata species, using the F95 pan-deimination antibody (MABN328, Merck) and the Catch and Release^®^v2.0 immunoprecipitation kit (Merck, UK). The F95 antibody specifically detects proteins modified by citrullination and has been developed against a decacitrullinated peptide [61], and found to cross-react with a number of species across phyla [23,24,25,26,27,28,36,52,53,54]. F95 enrichment was performed from a pool of approximately 200 parasites per species (the pool contained different gamogonic stages) at 4 °C overnight, using a rotating platform. Elution of the deiminated (F95-bound) proteins from the columns was performed according to the manufacturer’s instructions (Merck), and the protein eluate was thereafter diluted 1:1 in 2 × Laemmli sample buffer (BioRad, Watford, UK) and kept frozen at −20 °C until further use for SDS-PAGE analysis and in-gel digestion for LC-MS/MS analysis, as described below.

### 2.5. Western Blotting Analysis

For Western blotting, SDS-PAGE was carried out on the alveolate protein extracts. Samples were diluted 1:1 in denaturing 2 × Laemmli sample buffer (containing 5% beta-mercaptoethanol, BioRad) and heated for 5 min at 100 °C. Protein separation was carried out at 165 V for 50 min, using 4–20% gradient TGX gels (BioRad), followed by Western blotting at 15 V for 1 h using a Trans-Blot^®^ SD semi-dry transfer cell (BioRad). Membranes were stained with PonceauS (Sigma) to assess even protein transfer and then blocked with 5% bovine serum albumin (BSA, Sigma) in Tris buffered saline (TBS) containing 0.1% Tween20 (BioRad; TBS-T) for 1 h at room temperature. Primary antibody incubation was carried out overnight at 4 °C on a shaking platform using anti-human PAD2 antibody (anti-PAD2, ab50257, Abcam; diluted 1/1000 in TBS-T), for detection of putative PAD protein homologues, due to PAD2 being considered the most conserved PAD isozyme and the anti-human PAD2 antibody was previously shown to cross-react with PADs across taxa [23,24,25,26,27,28,29,36,54,55,62,63]. Deiminated histone H3 was tested using the citH3 antibody (Abcam, ab5103, diluted 1/1000 in TBS-T), also previously identified to cross react with a range of taxa [23,24,25,26,62,63]. The nitrocellulose membranes were washed following primary antibody incubation at RT in TBS-T for 3 × 10 min and thereafter incubated with HRP-conjugated secondary antibodies (anti-rabbit IgG (BioRad) diluted 1/3000 in TBS-T), for 1 h at RT. The membranes were washed for 5 × 10 min in TBS-T and digitally visualised, using enhanced chemiluminescence (ECL, Amersham, Little Chalfont, UK) in conjunction with the UVP BioDoc-ITTM System (Thermo Fisher Scientific, Hemel Hempstead, UK).

### 2.6. Silver Staining

SDS-PAGE (using 4–20% gradient TGX gels, BioRad) was carried out under reducing conditions for the total protein extracts as well as the F95-enriched protein eluates from both alveolate species. The gels were silver stained according to the manufacturer’s instructions, using the BioRad Silver Stain Plus Kit (1610449, BioRad, UK).

### 2.7. LC-MS/MS (Liquid Chromatography with Tandem Mass Spectrometry) Analysis of F95 Enriched Proteins

Liquid chromatography with tandem mass spectrometry (LC-MS/MS) was carried out to identify deiminated protein candidates from the two alveolates under study. For F95 enrichment, a pool of approximately 200 parasites was used, per species, and this pool contained various gamogonic stages per species. F95 enrichment was performed according to previously described methods in other taxa [36,53]. LC-MS/MS analysis was carried out following in–gel digestion, with the F95-enriched protein preparations (diluted 1:1 in 2 × Laemmli buffer and boiled for 5 min at 100 °C) run 0.5 cm into a 12% TGX gel (BioRad). The concentrated protein band (containing the whole F95 eluate) was excised, trypsin digested and subjected to proteomic analysis using a Dionex Ultimate 3000 RSLC nanoUPLC (Thermo Fisher Scientific Inc, Waltham, MA, USA) system in conjunction with a QExactive Orbitrap mass spectrometer (Thermo Fisher Scientific Inc., Waltham, MA, USA), performed by Cambridge Proteomics (Cambridge, UK), as previously described [27,29,36]. The data was processed post-run, using Protein Discoverer (version 2.1., Thermo Scientific) and MS/MS data were converted to mgf files which were submitted to the Mascot search algorithm (Matrix Science, London, UK) to identify deiminated protein hits. Search for F95 enriched proteins from both species was conducted against a common UniProt database against Alveolata CCP_Alveolata Alveolata_20201028 (1052932 sequences; 627,698,753 residues). An additional search was conducted against a common contaminant database (cRAP 20190401; 125 sequences; 41,129 residues). The fragment and peptide mass tolerances were set to 0.1 Da and 20 ppm, respectively, and the significance threshold value was set at of *p* < 0.05 and a peptide cut-off score of 48 was applied for the common Alveolata database (carried out by Cambridge Proteomics, Cambridge, UK).

### 2.8. Protein–Protein Interaction Network Analysis

To predict and identify putative protein–protein interaction networks associated with the deiminated proteins from the two alveolates, STRING analysis (Search Tool for the Retrieval of Interacting Genes/Proteins; https://string-db.org/, accessed on 15 February 2021) was performed. Protein networks were generated based on protein names and applying the function of “search multiple proteins” in STRING (https://string-db.org/, accessed on 15 February 2021). For a representative choice of database for Alveolata, *Toxoplasma gondii* was selected, as no species-specific protein databases are available for the two species under study in STRING. Networks were therefore built representative of Alveolata (with *T. gondii* showing most homology protein hits). Parameters applied in STRING were as follows: “basic settings” and “medium confidence”. Colour lines connecting the nodes represent the following evidence-based interactions for the network edges: “known interactions” (these are based on experimentally determined curated databases), “predicted interactions” (these are based on gene neighbourhood, gene co–occurrence, gene fusion, via text mining, protein homology or co–expression). Gene ontology network clusters for the deiminated protein networks were assessed in STRING and are highlighted by colour coding (see the corresponding colour code keys showing the individual nodes and connective lines within each figure).

### 2.9. Phylogenetic Comparison of Alveolata PAD Homologues

Previously reported Alveloata ADI protein sequences from two representative alveolates: *Gregarina niphandrodes* (XP_011128800.1) and *Babesia ovata* (XP_028866800.1), were aligned with human PAD isozyme sequences PAD1 (NP_037490.2), PAD2 (NP_031391.2), PAD3 (NP_057317.2), PAD4 (NP_036519.2), and PAD6 (NP_997304.3), as well as with bovine (*Bos taurus*) PAD1 (NP_001094742.1), PAD2 (NP_001098922.1), PAD3 (XP_010800991.1), PAD4 (NP_001179102.1) and PAD6 (XP_002685843.1), as well as *Giardia intestinalis* ADI (AAC06116.1) and teleost fish (sea bass—*Dicentrarchus labrax*) PAD (CBN80708.1), using Clustal Omega (https://www.ebi.ac.uk/Tools/msa/clustalo/, accessed on 15 February 2021). A neighbour-joining phylogeny tree was constructed.

## 3. Results

### 3.1. PAD Protein Homologue and Deiminated Protein Detection in Alveolates

**Figure 1:**
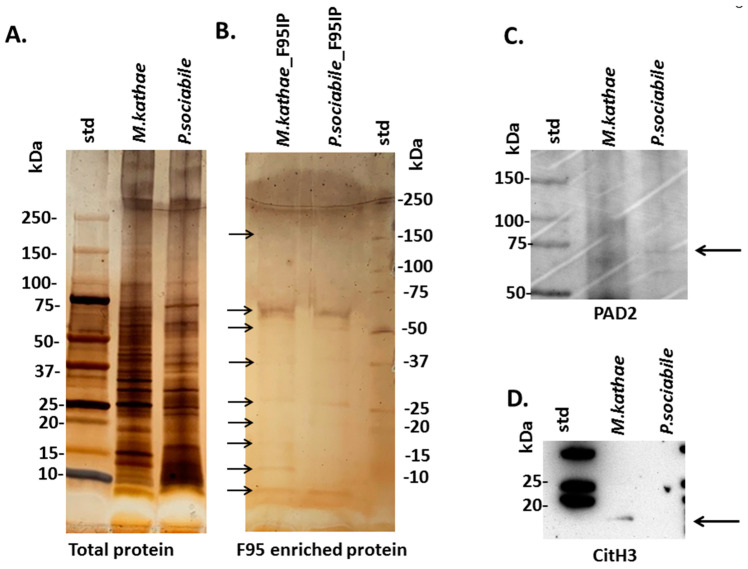
Alveolata PAD/ADI and deiminated protein identification. (**A**,**B**): silver stained SDS-PAGE gel (4–20% gradient TGX gel), showing total protein extraction (**A**) and F95-enriched fractions (**B**) from the two alveolate species (arrows point at F95 enriched protein bands observed, with some differences between the two species; the F95 eluate was further subjected to LC-MS/MS analysis for identification of deiminated protein candidates). (**C**). Western blotting analysis for PAD homologues in Alveolata, using the anti-human PAD2 antibody (see arrow for expected molecular weight size between 70–75 kDa, more clearly observed in *P. sociabile* than in *M. kathae*, where a smudged band between 50–100 kDa is observed). (**D**). CitH3 detection in both alveolates shows some positive reaction with *M. kathae*, but negligible with *P. sociabile*, possibly due to low protein load. The protein standard (std) is indicated in kilodaltons (kDa).

Total protein isolated form the two alveolates showed some differences in the protein pattern as assessed by SDS-PAGE and silver staining (Figure 1A). For assessment of putative deiminated proteins in the alveolates, F95-enriched protein fractions were separated by SDS-PAGE and silver stained, revealing protein bands in sizes ranging between 15–250 kDa with some differences in banding patterns between the two Alveolata (see arrows in Figure 1B); notably, as expected, the deiminated protein yield was low compared with total proteins detected. The deiminated (F95-enriced) proteins were further subjected to proteomic analysis for protein identification (Section 3.3). Anti-human PAD2 specific antibody was used for the assessment of a putative PAD protein homologue in alveolates, based on cross-reaction, using Western blotting. A faint positive protein band at an expected approximate 70–75 kDa size was identified in the chromerid *P. sociabile* (see arrow in Figure 1C), while in the apicomplexan *M. kathae*, some faint reaction for a smudged band in a 50–100 kDa region was observed (Figure 1C). The presence of deiminated histone H3 was also assessed by Western blotting, showing some reaction at the expected band size (approximately 17 kDa) with *M. kathae*, while reaction with *P. sociabile* was negligible, very possibly due to low protein load (Figure 1D).

**Figure 2:**
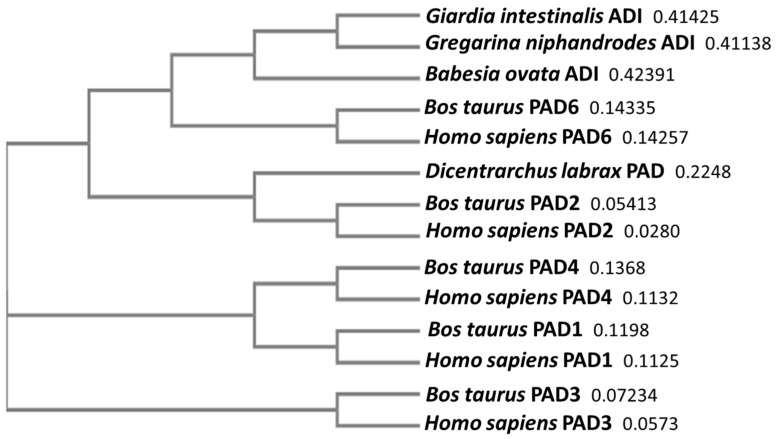
A neighbour-joining phylogeny tree for Alveolata ADI/PAD homologues. Known ADI/PAD protein sequences from two representative alveolates (*Gregarina niphandrodes* and *Babesia ovate*—as protein ADI sequences that have been reported in these species —show closest similarity to the known parasite ADI (*Giardia intestinalis*). The closest homology was then found with mammalian PAD6 and PAD2, followed by teleost PAD (*Dicentrarchus labrax* shown as an example). The tree includes all five human (*Homo sapiens*) and bovine (*Bos taurus*) PAD isozymes (PAD1, 2, 3, 4 and 6, respectively) to represent mammalian PADs (the numbers next to the species names represent a measure of support for the node).

In order to position Alveolata ADI/PAD in phylogeny, a neighbour-joining tree was constructed for previously reported Alveloata ADI protein sequences from two representative alveolates: *Gregarina niphandrodes* (XP_011128800.1) and *Babesia ovata* (XP_028866800.1), in comparison with mammalian (human and bovine) PAD isozyme sequences PAD1, 2, 3, 4, 6 and teleost PAD, as well as *Giardia intestinalis* ADI. Alveolata ADI/PAD revealed the closest similarity to *Giardia* ADI and mammalian PAD6, followed by teleost PAD and mammalian PAD2 (Figure 2).

### 3.2. Histological Analysis of PAD and Protein Deimination in Alveolata

#### 3.2.1. Peptidylarginine Deiminase (PAD)

**Figure 3:**
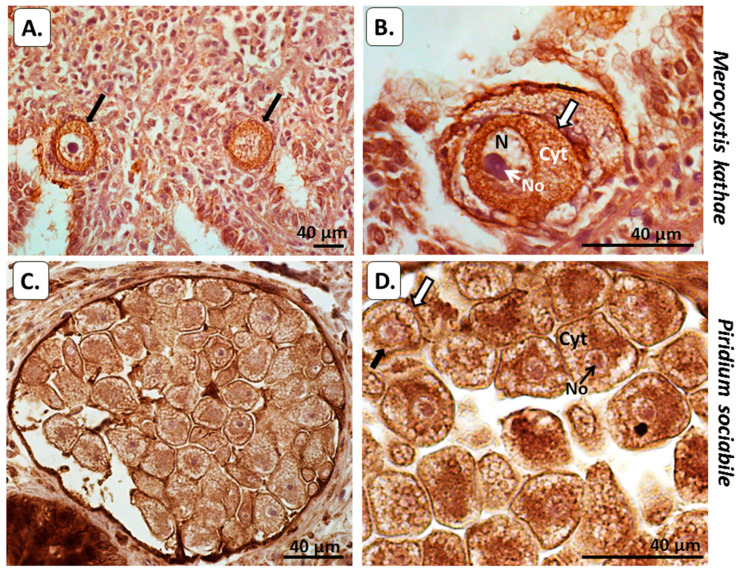
Peptidylarginine deiminase (PAD) in the two alveolates under study. (**A**,**B**) *M. kathae* in the kidney of a common whelk. (**A**) Two macrogamonts with strong PAD positive reaction detected in the cytoplasm (cyt) and in the cell membrane (black arrows). Surrounding host tissue (whelk kidney) is also PAD positive as expected. (**B**) Higher magnification of a representative macrogamont with prominent nucleus (N) and nucleolus (No); cell membrane highlighted by white arrow–strong positive PAD reaction is observed in *M. kathae* cytoplasm. (**C**,**D**) *P. sociabile* in the whelk foot. Numerous, encysted parasite bodies of *P. sociabile* (**C**) and several of these forms at higher magnification (**D**). The figures show strong PAD positive staining in the cytoplasm (cyt), the plasma (white arrow) and nuclear membrane (black arrow), and some low reaction in the nucleolus (No). The fibromuscular host tissue (whelk) is positive for PAD, as would be expected as PAD is strongly expressed in muscle. Scale bar is indicated in each figure and is 40 µm.

In *M. kathae*, a strong positive reaction for PAD protein (using the anti-human PAD2 antibody) was seen in the cytoplasm and in the outer membrane. The surrounding kidney tissue of the host was strongly positive for PAD, as expected (Figure 3A,B). In *Piridium sociabile*, PAD positive staining was detected in the cytoplasm, as well as some positive reaction in the surrounding molluscan host muscle (Figure 3C,D).

#### 3.2.2. Total Deiminated Protein Detection Using Pan-Citrulline Antibody F95

**Figure 4:**
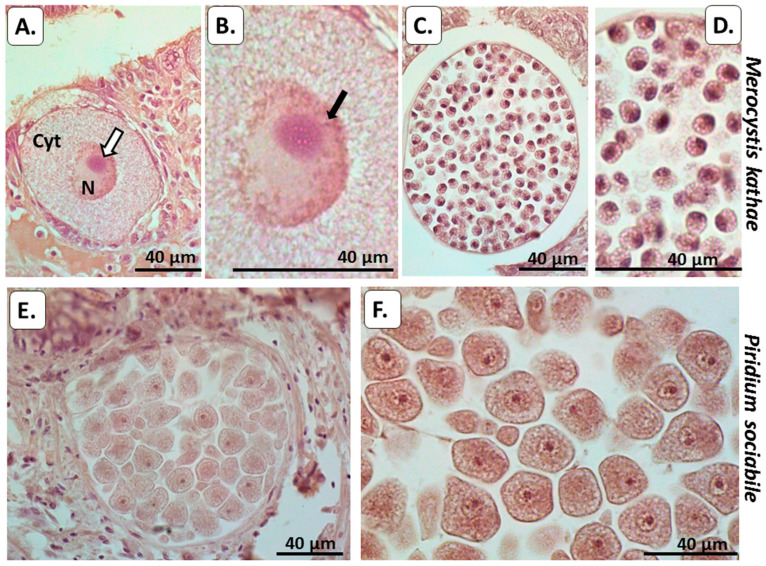
Deiminated proteins detected by pan-citrulline F95 antibody in the two alveolate species. (**A**–**D**) A whelk kidney infected with *Merocystis kathae*: (**A**) Mature macrogamont of *M. kathae* with a prominent nucleus (N) and nucleolus (white arrow). (**B**) Higher magnification of a mature macrogamont showing the positive F95 detection in more details. In addition to the nuclear membrane, the positive F95 reaction seems to extend into the nucleus itself (arrow). (**C**) An immature *M. kathae* oocyst with numerous sporoblasts. (**D**) Sporoblasts shown at higher magnification, showing positive F95 reaction in the cytoplasm of the sporoblasts. (**E**) Numerous encysted parasite bodies of *P. sociabile* in the whelk foot. (**F**) Higher magnification, showing several parasite bodies with obvious F95-positive reaction in the cytoplasm, nucleus and nucleolus. The surrounding host tissues (whelk kidney and foot) also show some F95 positive. Scale bar is indicated in each figure and is 40 µm.

In *M. kathae*, some faint F95 positive was detected in cytoplasm and outer membrane, as well as in the surrounding kidney host tissue (Figure 4A–D). F95 positive reaction, identifying total deiminated/citrullinated proteins was seen in *P. sociabile* in the cytoplasm. Some F95 positive was also detected in the host (whelk) muscle tissue as would be expected (Figure 4E,F).

#### 3.2.3. Deiminated/Citrullinated Histone H3

**Figure 5:**
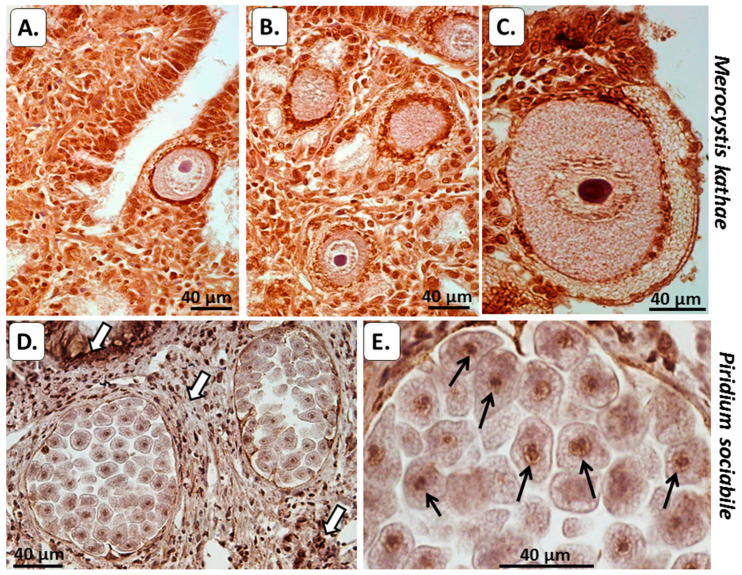
Deiminated/citrullinated histone H3 (CitH3) in the two alveolates under study. (**A**–**C**) Whelk kidney infected with *M. kathae*. Strong host-reaction indicative of NET/ETosis is observed by the increased CitH3 staining in tissue surrounding *M. kathae* infection. In *M. kathae*, strong CitH3 positive is observed in the outer membrane, cytoplasm, nucleus and nucleolus. (**D**) Two *P. sociabile* cysts in a whelk foot. Quite strong reaction is evident in the surrounding host tissues, both in the whelk fibromuscular connective tissue as well as the epithelial layer (white arrows). (**E**) Higher magnification of several parasite bodies showing moderate CitH3 reaction in the cytoplasm and strong reaction in the nuclear membrane and the nucleolus (black arrows). Scale bar is indicated in each figure and is 40 µm.

Deiminated/citrullinated histone H3 (CitH3) was strongly detected in the outer membrane of *M. kathae* and was also strongly detected in surrounding host tissue infected with *M. kathae*, indicative of NET/ETosis (Figure 5). Histone H3 deimination (CitH3) was strongly detectable in *P. sociabile* nucleus. Strong CitH3 positive was also detected in the surrounding host (whelk) fibromuscular connective tissue, indicative of NET/ETosis around infected areas.

### 3.3. LC-MS/MS Analysis of Deiminated Protein Targets in Alveolata

**Figure 6:**
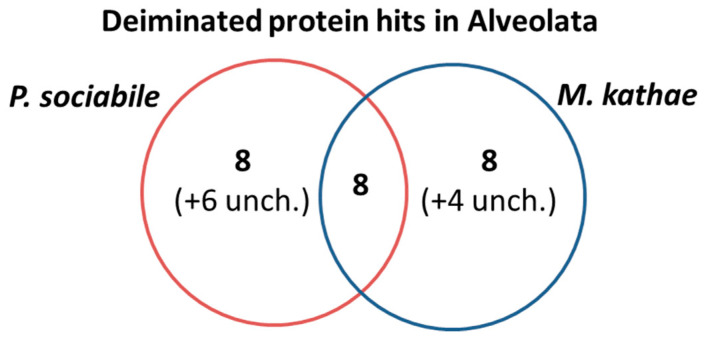
Deiminated protein hits identified in *P. sociabile* and *M. kathae*. Overall 8 deimination hits overlapped with the two Alveolata under study, while 8 hits were specific to *P. sociabile* (with further 6 uncharacterised hits), and 8 hits were specific to *M. kathae* (with further 4 uncharacterised hits). See Table 1 and Table 2 for the individual hits.

The identification of deiminated proteins in the two Alveolata under study (from a pool of approximately *n* = 200 parasites per species) was carried out following F95-enrichment using LC-MS/MS analysis. Species-specific protein hits with the individual species, as well as hits with other Alveolata were identified using the UniProt Alveolata database (Table 1 and Table 2; see Supplementary Tables S1 and S2 for full details on all peptide hits). Overall, 14 protein hits were specific to *P. sociable* (whereof 6 were uncharacterised hits) and 12 were specific to *M. kathae* (whereof 4 were uncharacterised hits). A further 8 hits were shared between both species as outlined in Table 1 and Table 2 and the Venn diagram in Figure 6.

### 3.4. Protein–Protein Interaction Network Identification of Deiminated Proteins in Alveolata

**Figure 7:**
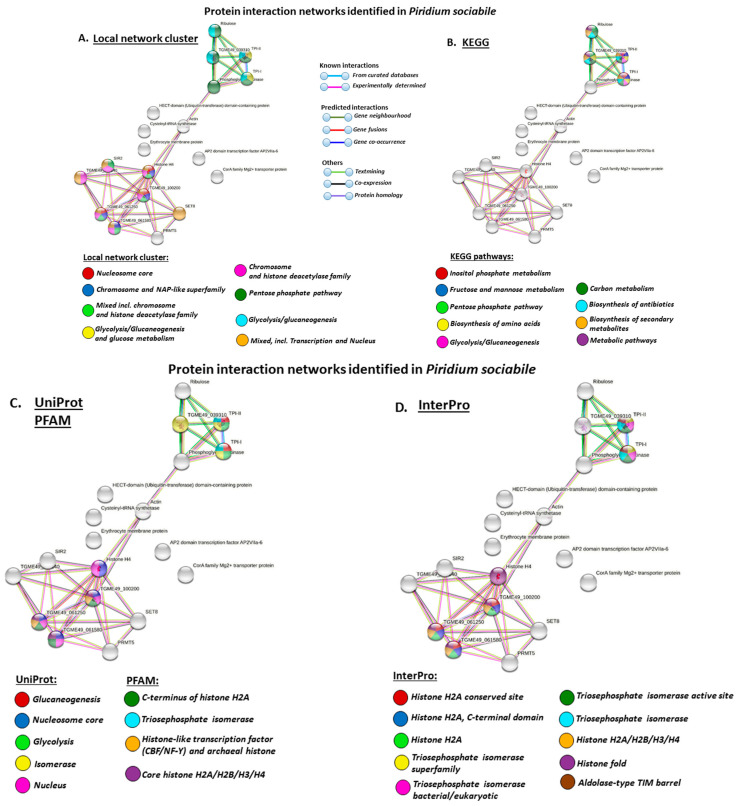
Local Search Tool for the Retrieval of Interacting Genes/Proteins (STRING) network clusters, KEGG pathways and protein domains identified for deiminated proteins in *Piridium sociabile*. Protein–protein interaction network identified in *P. sociabile* based on protein identifiers from *T. gondii*. PPI enrichment *p*-value: 0.000203. (**A**). Local network clusters are highlighted with the coloured nodes. (**B**). KEGG pathways are highlighted with the coloured nodes. (**C**). UniProt keywords and PFAM protein domains are highlighted with the coloured nodes. (**D**). Protein domains and features are highlighted with the coloured nodes. Colour coding for network nodes and interaction lines is included in the figure.

**Figure 8:**
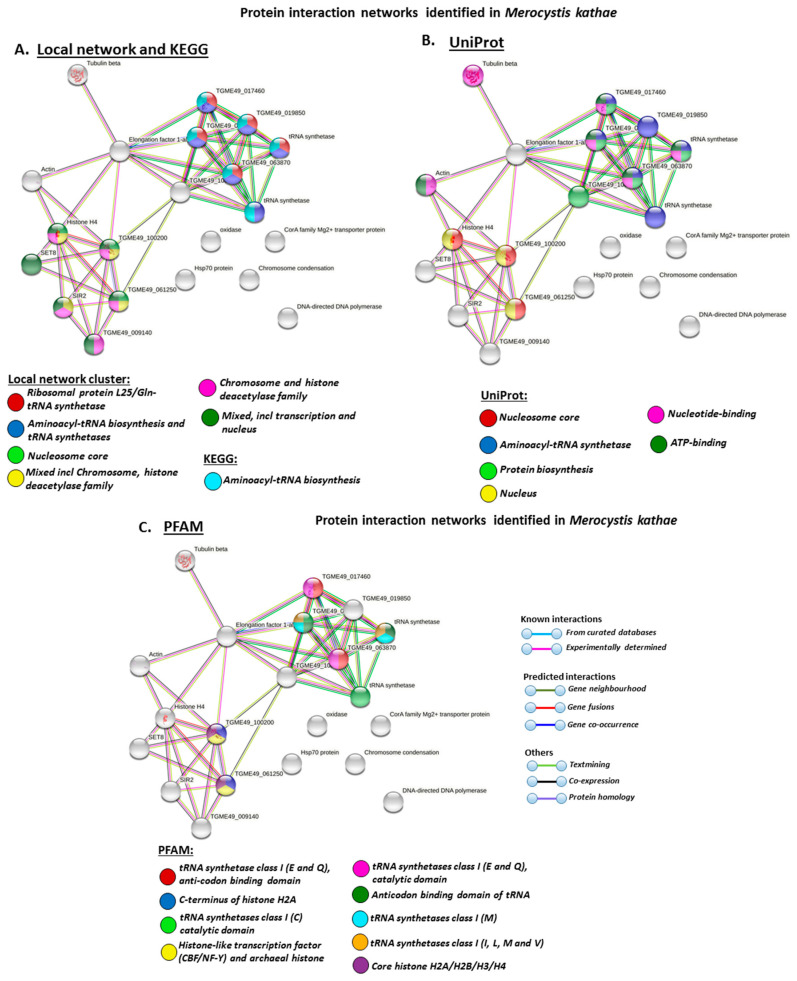

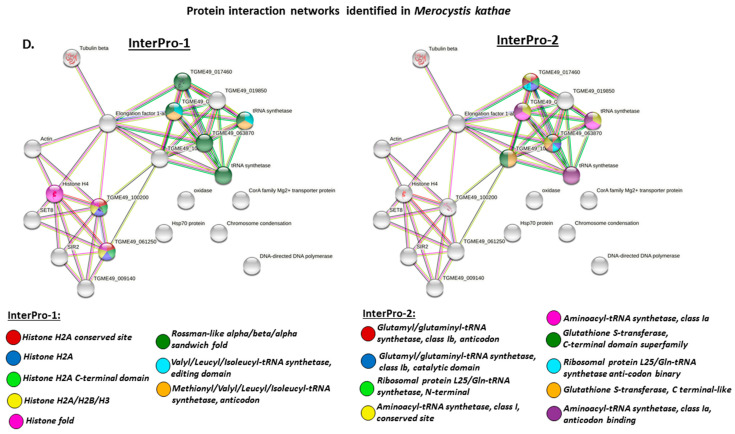
Local STRING network clusters and protein domains identified for deiminated proteins in *Merocystis kathae*. Protein–protein interaction network identified in *M. kathae* based on protein identifiers from *T. gondii*. PPI enrichment *p*-value: 0.000138. (**A**). Local network clusters and KEGG pathways are highlighted with the coloured nodes. (**B**). UniProt keywords are highlighted with the coloured nodes. (**C**). PFAM protein domains are highlighted with the coloured nodes. (**D**). Protein domains and features (Interpro-1 and -2) are highlighted with the coloured nodes. Colour coding for network nodes and interaction lines is included in the figure.

For the prediction of protein–protein interaction networks of the deimination candidate proteins identified in the alveolates under study, the protein names were submitted to STRING (Search Tool for the Retrieval of Interacting Genes/Proteins) analysis (https://string-db.org/, accessed on 15 February 2021). Protein interaction networks were based on known and predicted interactions and represent all deiminated proteins identified in the different alveolates and their interaction partners present in the STRING database, based on networks for *Toxoplasma gondii* as a representative Alveolata (Apicomplexa) species (this had overall the maximum hits with the corresponding species specific proteins, although all proteins were not always found in the *T. gondii* database), as protein identifiers for the individual species was not available in STRING (Figure 7 and Figure 8).

## 4. Discussion

The current study assessed PAD homologues and post-translationally deiminated protein signatures in two alveolates, providing novel insights into mechanisms in metabolism, epigenetic regulation and host–pathogen interactions. This highlights putative roles for post-translational modifications in the functional diversification of conserved protein pathways throughout phylogeny.

This is the first description of deiminated protein products in parasitic alveolates, while PAD homologues/arginine deiminase (ADI) protein sequences have been reported in two apicomplexan, *Babesia* spp. and *Gregarina* spp. The genus *Babesia* is comprised of over 100 species of tick-borne parasites that infect erythrocytes in a range of vertebrate hosts, affecting livestock, wild and domestic animals worldwide, as well as occasionally humans, causing babesiosis [64,65,66]. *Gregarina niphandrodes* is an early diverging apicomplexan closely related to *Cryptosporidium* spp. [67]. The current study identified that PAD/ADI are also present in the chromerid *P. sociabile* and the apicomplexan *M. kathae*, the two Alveolata under study here, and provides the first evidence that alveolates are active in producing citrullinated/deiminated protein products which may regulate critical pathways in Alveolata metabolism and gene regulation.

Using immunohistochemical detection for PAD and deiminated protein products for the two Alveolata in host tissue, evidence was found for both PAD/ADI homologue and protein deimination/citrullination in cytoplasmic and nuclear compartments of the Alveolata. Furthermore, host–pathogen interactions by means of histone H3 citrullination/deimination, indicative of NET/ETosis, were also strongly observed in the host tissue for both alveolates. In *M. kathae*, strong reaction with the PAD antibody was observed in the cytoplasm as well as in the plasma and nuclear membranes. PAD was also positive in surrounding kidney tissue of the Mollusca (whelk) host (Figure 3); which was expected, as the PAD2 antibody cross reacts with a number of species, including Mollusca [29], and is the most ubiquitously expressed PAD [21], including in kidney and muscle [23,24]. In *M. kathae*, some faint F95 positive staining was detected in the cytoplasm nuclear membrane of macrogamonts and the cytoplasm of sporoblasts (immature sporozoites/infective life stage). Some F95 reaction was also observed in the surrounding kidney host tissue, which may relate to changes in protein deimination in response to infection (Figure 4A–D). Deiminated/citrullinated histone H3 positive staining was strongly detected in the outer membrane and nucleolus of *M. kathae* and was also strongly detected in the surrounding host fibromuscular connective tissue infected with this apicomplexan, indicative of NET/Etosis as part of the host defence mechanism against the pathogen (Figure 5A–C).

In *P. sociabile*, PAD was detected in the cytoplasm, plasma- and nuclear membrane and some faint positive was seen in the nucleolus. PAD-positive reaction was also observed in the surrounding Mollusca host´s fibromuscular tissue (Figure 3C,D). Indeed, muscle is well known to be rich in PAD protein [21,23,24], and a recent report has furthermore identified PAD homologue and deiminated proteins in Mollusca [29]. Furthermore, some F95 positive staining was also detected in *P. sociabile* cytoplasm, nucleus and nucleolus (Figure 4E,F), supporting the presence of deiminated proteins in these sites. Using the deiminated/citrullinated histone H3 (CitH3) antibody, a strong positive response was seen in the nucleus and nucleolus of *P. sociabile*, which is indicative of epigenetic regulation in this chromerid parasite (Figure 5D,E). The detection of CitH3 in the Alveolata by histological staining does correlate with findings from proteomic analysis of the deiminated proteins from both Alvelolata, where deimination hits included core histones, albeit only histone H4 was identified as a certain hit, while further unidentified hits remain to be revealed. The deimination of histones relates to a number of gene regulatory GO pathways, as listed below.

Using proteomic analysis in conjunction with F95 enrichment for pan-citrullination, 14 deimination protein hits were specific to *P. sociable* (whereof 6 were uncharacterised hits) and 12 hits were specific to *M. kathae* (whereof 4 were uncharacteristed hits). A further 8 deimination protein hits were identified to be shared between both species. Protein hits identified as common deimination targets in both Alveolata species under study were GFP-like fluorescent chromoprotein FP506, GFP, Actin (and actin II), Histone H4, AIG1-type *G* domain-containing protein, Cysteinyl-tRNA synthetase and CorA family Mg^2+^ transporter protein. Deiminated protein hits identified only in *P. sociabile* were Ribulose-1,5-bisphosphate carboxylase/oxygenase large subunit, Beta-helix domain-containing protein, AP2 domain transcription factor AP2VIIa-6, Pep3-Vps18 domain-containing protein, Phosphoglycerate kinase, HECT-domain (Ubiquitin-transferase) domain-containing protein, Protein-serine/threonine phosphatase and Erythrocyte membrane protein 1 (PfEMP1). Deiminated protein hits specific to *M. kathae* were Elongation factor 1-alpha, Alternative oxidase, Ribulose bisphosphate carboxylase, Hsp70 protein, DNA-directed DNA polymerase, P-loop containing nucleoside triphosphate hydrolase, chromosome condensation regulator repeat protein and Tubulin beta chain.

Using GO analysis for protein–protein interaction pathways relating to the deiminated protein candidates identified in both species, the local network clusters identified for the deiminated proteins in *P. sociabile* were the nucleosome core, chromosome and NAP-like superfamily, chromosome and histone deacetylase family, chromosome and histone deacetylase family, transcription and nucleus, Pentose phosphate pathway, glycolysis/glucaneogenesis and glucose metabolism. KEGG pathways identified for the deiminated proteins in *P. sociabile* were metabolic pathways, inositol phosphate metabolism, fructose and mannose metabolism, pentose phosphate pathway, biosynthesis of amino acids, glycolysis/gluconeogenesis, carbon metabolism, biosynthesis of antibiotics, and biosynthesis of secondary metabolites. UniProt keywords related to gluconeogenesis, glycolysis, isomerase, nucleus and nucleosome core, while PFAM domains were C-terminus of histone H2A, histonelike transcription factor (CBF/NF-Y) and archaeal histone, Core histone H2A/H2B/H3/H4 and Triosephosphate isomerase. InterPro keywords also highlighted histones as well as Triosephosphate isomerase and Aldolase-type TIM barrel to be enriched for protein deimination.

In *M. kathae*, local network clusters for deiminated proteins included ribosomal protein L25/Gln-tRNA synthetase, aminoacyl-tRNA biosynthesis and tRNA synthetases, nucleosome core, mixed incl chromosome, histone deacetylase family, chromosome and histone deacetylase family, transcription and nucleus. KEGG pathways for deiminated proteins highlighted aminoacyl-tRNA biosynthesis. UniProt keywords for deiminated proteins related to nucleosome core, aminoacyl-tRNA synthetase, protein biosynthesis, nucleus, nucleotide-binding and ATP-binding. PFAM domains for deiminated proteins in *M. kathae* related to tRNA synthetase, anticodon binding domain of tRNA, histonelike transcription factor (CBF/NF-Y) and archaeal histone and core histone H2A/H2B/H3/H4.

While GO terms for deiminated proteins were found to be common for some pathways in both Alveolata species under study, particularly with relation to gene regulatory pathways such as histones, there were far more pathways relating to metabolism linked to deiminated proteins in *P. sociabile*, compared with in *M. kathae*. Furthermore, in *M. kathae* ribosomal pathways and tRNA biosynthesis are highlighted for deiminated proteins as well as ATP-binding, but this was not identified in *P. sociabile*. Therefore, while some key pathways are in common for deiminated proteins in both Alveolata under study here, species-specific differences are apparent.

Histones are well known to be targets of deimination for gene regulatory and epigenetic changes in a range of human pathologies, as well as in developmental processes [40,41,68]. In Apicomplexa, the roles for epigenetic regulation in the life cycle and host–pathogen interactions have received considerable attention [69,70,71,72,73,74,75], while deimination remains to be studied in this context and is here identified and described for the first time in Alveolata species. It must be noted that in the LC-MS/MS analysis, only histone H4 came up as a citrullinated/deiminated candidate for the alveloates under study, while a number of histone pathways were enriched in the protein-interaction network analysis. As the F95 antibody is a pan-citrullin antibody and not specifically directed against histone deimination, the number of histone candidates in the proteomic analysis may be underestimated; furthermore it must be considered that a number of uncharacterised hits was also present. Due to CitH3 being the only commercially available antibody for a deiminated histone (and positive reaction for F95 in the Alveolata nucleus may be indicative of histone deimination), the CitH3 antibody was used here to assess putative histone H3 deimination in the parasites. Furthermore, CitH3 is a valuable marker indicative of NET/ETosis in host responses against pathogens, and did show a strong reaction with the respective host tissues surrounding the parasites.

Besides the modulation of a range of pathways described here, interestingly recent approaches in *Giardia intestinalis* using pharmacological PAD inhibitor treatment showed a significant reduction of parasite adhesion to human intestinal cells. This was in part attributed to regulatory roles of PADs in extracellular vesicle (EV) release, which are membrane bound vesicles released from cells (parasite and host) and participate in host–pathogen interactions. It was shown that PAD inhibition resulted in decreased EV release from *G. intestinalis* and contributed to hindering parasite adhesion to the host [31]. PAD inhibitors have also been shown to modulate EV/MV release from bacteria and affect antibiotic resistance, pointing to a conserved pathway for this enzyme family in EV modulation throughout phylogeny [33]. Therefore, in what way ADI/PAD in alveolates, e.g., apicomplexan and chromerids, may further play roles in EV regulation in host–pathogen interactions remains subject to further studies.

It must be noted that the analysis of deiminated proteins included all gamogonic stages of *M. kathae* and therefore it remains to be further investigated how deimination may play roles in their different life cycle stages. This also has to be considered in relation to the life cycle stages in the scallop intermediate host. Regarding *P. sociabile*, although described in the 1930s, much remains to be elucidated about the life cycle of this species and the role for deimination in the life cycle of this phylogenetically unique chromerid, which provides an evolutionary link between free living/symbiotic photosynthetic organisms and parasites.

Likewise of interest is that while all apicomplexans are considered parasitic and some are highly pathogenic, much wider roles for their function in host interactions have been suggested, including commensalism and mutualism [76]. Given the conserved status of PAD/ADI throughout phylogeny, the roles for the deimination regulation of the pathways involved in host–pathogen interaction and the coevolution of Alveolata may therefore be of considerable interest, particularly in the light of the various pathways identified here for deimination, which are linked to a wide range of metabolic processes, transcription and gene regulation. The presence of deiminated protein products in host tissue surrounding the parasites will also require further evaluation, including whether such deimination is part of the direct immune response of the host or whether the parasite ADI causes the deimination of the host proteins, aiding parasite immune evasion and causing tissue remodeling in the host. Such post-translational interaction between the parasite and the host may possibly contribute to mutualism and symbiosis, in addition to forming part of the host-defence.

An interesting observation is that alveolate PAD/ADI seems closer to human PAD6 than PAD2, which is generally considered the phylogenetically most conserved PAD, as seen with other species, including teleost fish where only one PAD exists, which is closest to mammalian PAD2. In mammals, PAD6 plays important roles in early development, embryo preimplantation and early embryonic arrest [77,78,79,80], affecting KEGG pathways of multicellular organism development, cell differentiation and oocyte differentiation [81]. It may be speculated, given the detrimental effects of some apicomplexan infections such as toxoplasmosis and malaria (*Plasmodium* spp.) on the developing foetus, disrupting foetal development [82], that the ADI of these parasites may affect the developmental processes normally regulated by human PADI6. This remains though a topic of further investigation.

This is the first study to assess protein deimination in Alveolata, including Chromerida, and contributes to current understanding of the phylogenetically conserved and differing roles of PADs/ADI in the regulation of pathways involved in immune, metabolic and epigenetic regulation across phylogeny. Future studies must, amongst other, evaluate deimination at the various gamogonic stages and stages of the parasites’ life cycles. Deimination regulated pathways may play hitherto under recognised roles in Alveolata life cycle and in host–pathogen interactions.

## 5. Conclusions

This study provides the first insights into regulatory roles for protein deimination in Alveolata, in relation to epigenetic regulation and putative roles in the parasite lifecycles, as well as in host–pathogen interactions. Much remains to be understood about the roles for PAD/ADI-mediated regulation parasite lifecycles, as well as in host–pathogen interactions, throughout the phylogeny tree. Furthermore, as the Alveolata are a complex and diverse group of pathogens, studies into novel molecular mechanisms, including post-translationally mediated processes such as deimination, may provide novel insights into their adaptability to different environments and hosts and host–pathogen coevolution. Future studies will aim at understanding PAD/ADI roles in *Piridium sociable* and *Merocystis kathae* life cycles and host–pathogen interactions, including in intermediate host species.

## Figures and Tables

**Table 1:** Deiminated proteins in *Piridium sociabile*, as identified by F95-enrichment in conjunction with LC-MS/MS analysis. Deiminated proteins were isolated by immunoprecipitation using the pandeimination F95 antibody. The resulting F95-enriched eluate was then analysed by LC-MS/MS and peak list files submitted to Mascot, using a common Alveolata database. Peptide sequence hits are listed, showing species-specific hits, number of sequences for protein hits and total score. Species hit names and phylum are indicated; a full list of protein sequence hits and peptides is further provided in Supplementary Table S1. Proteins highlighted in pink were deimination candidate hits only identified in *P. sociabile,* not in *M. kathae*.

Protein ID *Protein Name*	*Species Name*	Phylum	Matches (Sequences)	Total Score (*p* < 0.05) ^ⱡ^
**U6GSR1_EIMAC** *GFP-like fluorescent chromoprotein FP506*	*Eimeria acervulina*	Apicomplexa	18 (7)	378
**I1YZZ9_BABBO** *GFP-BSD*	*Babesia bovis*	Apicomplexa	18 (7)	
**B3SHQ6_9CILI** *Actin (Fragment)*	*Heterometopus palaeformis*	Ciliophora	2 (2)	129
**Q6 × 407_9SPIT** *Ribulose-1,5-bisphosphate carboxylase/oxygenase large subunit*	*Strombidium* sp.	Ciliophora	5 (3)	116
**A0A023B6S9_GRENI** *Histone H4*	*Gregarina niphandrodes*	Apicomplexa	3 (2)	111
**A0A0G4HXG8_9ALVE** *Beta_helix domain-containing protein*	*Chromera velia*	Chromerida	5 (2)	102
**A0A061DCJ4_BABBI** *Histone H4*	*Babesia bigemina*	Apicomplexa	8 (2)	83
**A0A1Q9DJ14_SYMMI** *Uncharacterised protein*	*Symbiodinium microadriaticum*	Dinoflagellata	1 (1)	65
**Q968T9_9SPIT** *Actin II (Fragment)*	*Diophrys* sp.	Ciliophora	1 (1)	61
**A0A1R2CHZ0_9CILI** *AIG1-type G domain-containing protein*	*Stentor coeruleus*	Ciliophora	7 (1)	60
**A0A0G4EPG2_VITBC** *Uncharacterised protein*	*Vitrella brassicaformis*	Chromerida	2 (1)	59
**U6LWU9_EIMMA** *Cysteinyl-tRNA synthetase, putative*	*Eimeria maxima*	Apicomplexa	1 (1)	55
**Q5CUG1_CRYPI** *Uncharacterised protein*	*Cryptosporidium parvum*	Apicomplexa	2 (2)	55
**A0A0J9SEN8_PLAVI** *Uncharacterised protein*	*Plasmodium vivax India VII*	Apicomplexa	7 (2)	54
**A0A139XZM4_TOXGO** *AP2 domain transcription factor AP2VIIa-6*	*Toxoplasma gondii ARI*	Apicomplexa	8 (2)	53
**A0A1J1HE72_PLARL** *Pep3_Vps18 domain-containing protein*	*Plasmodium relictum*	Apicomplexa	1 (1)	53
**A0A086JH54_TOXGO** *CorA family Mg2+ transporter protein*	*Toxoplasma gondii*	Apicomplexa	8 (1)	53
**A0A1R2C5U2_9CILI** *Uncharacterised protein*	*Stentor coeruleus*	Ciliophora	1 (1)	52
**A0A0G4GAZ1_VITBC** *Phosphoglycerate kinase*	*Vitrella brassicaformis*	Chromerida	1 (1)	51
**Q22CS8_TETTS** *Uncharacterised protein*	*Tetrahymena thermophila (strain SB210)*	Ciliophora	1 (1)	50
**A0A2A9LZE3_9APIC** *HECT-domain (Ubiquitin-transferase) domain-containing protein*	*Besnoitia besnoiti*	Apicomplexa	1 (1)	48
**A0A023B8K1_GRENI** *Protein-serine/threonine phosphatase*	*Gregarina niphandrodes*	Apicomplexa	2 (1)	48
**A0A2P9DSP9_PLARE** *Erythrocyte membrane protein 1, PfEMP1*	*Plasmodium reichenowi*	Apicomplexa	2 (2)	48

^ⱡ^ Ions score is −10 * Log(P), where P is the probability that the observed match is a random event. Individual ion scores > 48 indicate identity or extensive homology (*p* < 0.05). Protein scores are derived from ions scores as a non-probabilistic basis for ranking protein hits.

**Table 2:** Deiminated proteins in *Merocystis kathae*, as identified by F95-enrichment in conjunction with LC-MS/MS analysis. Deiminated proteins were isolated by immunoprecipitation using the pandeimination F95 antibody. The resulting F95-enriched eluate was then analysed by LC-MS/MS and peak list files submitted to Mascot, using a common Alveolata database. Peptide sequence hits are listed, showing species-specific hits, number of sequences for protein hits and total score. Species hit names and phylum are indicated; a full list of protein sequence hits and peptides is further provided in Supplementary Table S2. Proteins highlighted in green were deimination candidate hits only identified in *M. kathae*, not in *P. sociabile*.

Protein ID *Protein Name*	*Species Name* Common Name	Phylum	Matches (Sequences)	Total Score (*p* < 0.05) ^ⱡ^
**U6GSR1_EIMAC** *GFP-like fluorescent chromoprotein FP506, related*	*Eimeria acervulina*	Apicomplexa	39 (16)	809
**A0A023B6S9_GRENI** *Histone H4*	*Gregarina niphandrodes*	Apicomplexa	3 (2)	136
**B3SHQ6_9CILI** *Actin (Fragment)*	*Heterometopus palaeformis*	Ciliophora	3 (3)	128
**A0A0G4EA45_VITBC** *Histone H4*	*Vitrella brassicaformis*	Chromerida	3 (2)	127
**A0A061DCJ4_BABBI** *Histone H4*	*Babesia bigemina*	Apicomplexa	4 (2)	121
**A0A0D9QNG9_PLAFR** *Elongation factor 1-alpha*	*Plasmodium fragile*	Apicomplexa	4 (3)	105
**C5LYZ2_PERM5** *Alternative oxidase, putative*	*Perkinsus marinus*	Perkinsozoa	2 (2)	82
**A0A0F7R4L8_LEPCH** *Ribulose bisphosphate carboxylase large chain*	*Lepidodinium chlorophorum*	Dinoflagellata	6 (2)	77
**Q9GRE8_TOXGO** *Hsp70 protein*	*Toxoplasma gondii*	Apicomplexa	18 (1)	64
**A0A0V0QLA9_PSEPJ** *Uncharacterised protein*	*Pseudocohnilembus persalinus*	Ciliophora	2 (2)	64
**A0A1R2CHZ0_9CILI** *AIG1-type G domain-containing protein*	*Stentor coeruleus*	Ciliophora	5 (1)	60
**A0A086JH54_TOXGO** *CorA family Mg2+ transporter protein*	*Toxoplasma gondii*	Apicomplexa	6 (1)	58
**A0A2C6KZC4_9APIC** *Uncharacterised protein*	*Cystoisospora suis*	Apicomplexa	2 (2)	58
**Q968T9_9SPIT** *Actin II (Fragment)*	*Diophrys* sp.	Ciliophora	1 (1)	57
**A0A0F7UJI8_NEOCL** *DNA-directed DNA polymerase*	*Neospora caninum (strain Liverpool)*	Apicomplexa	2 (2)	56
**A0A2P9CCB1_9APIC** *P-loop containing nucleoside triphosphate hydrolase*	*Plasmodium gaboni*	Apicomplexa	2 (2)	54
**A0A2C6LD60_9APIC** *Chromosome condensation regulator repeat protein*	*Cystoisospora suis*	Apicomplexa	3 (2)	53
**G0QSP8_ICHMG** *Uncharacterised protein*	*Ichthyophthirius multifiliis (strain G5)*	Ciliophora	2 (1)	53
**A0A060BG26_9CILI** *Tubulin beta chain*	*Stentor coeruleus*	Ciliophora	2 (2)	52
**A0A1Q9C0L9_SYMMI** *Uncharacterised protein*	*Symbiodinium microadriaticum*	Dinoflagellata	4 (1)	51
**U6LWU9_EIMMA** *Cysteinyl-tRNA synthetase, putative*	*Eimeria maxima*	Apicomplexa	1 (1)	50

^ⱡ^ Ions score is −10 * Log(P), where P is the probability that the observed match is a random event. Individual ion scores > 48 indicate identity or extensive homology (*p* < 0.05). Protein scores are derived from ions scores as a non-probabilistic basis for ranking protein hits.

## Data Availability

Data are contained within the article and supplementary material.

## Supplementary Materials

The following are available online at https://www.mdpi.com/2079-7737/10/3/177/s1, Table S1: Full LC-MS/MS analysis of F95-enriched proteins in *P. sociabile*, Table S2: Full LC-MS/MS analysis of F95-enriched proteins in *M. kathae*.
