# Supplementary figures and images for: Peptidylarginine Deiminase (PAD) and Post-Translational Protein Deimination—Novel Insights into Alveolata Metabolism, Epigenetic Regulation and Host–Pathogen Interactions
Source: Biology (Basel). 2021 Feb 26;10(3):177. doi: 10.3390/biology10030177 (PMC7996758; doi:10.3390/biology10030177)

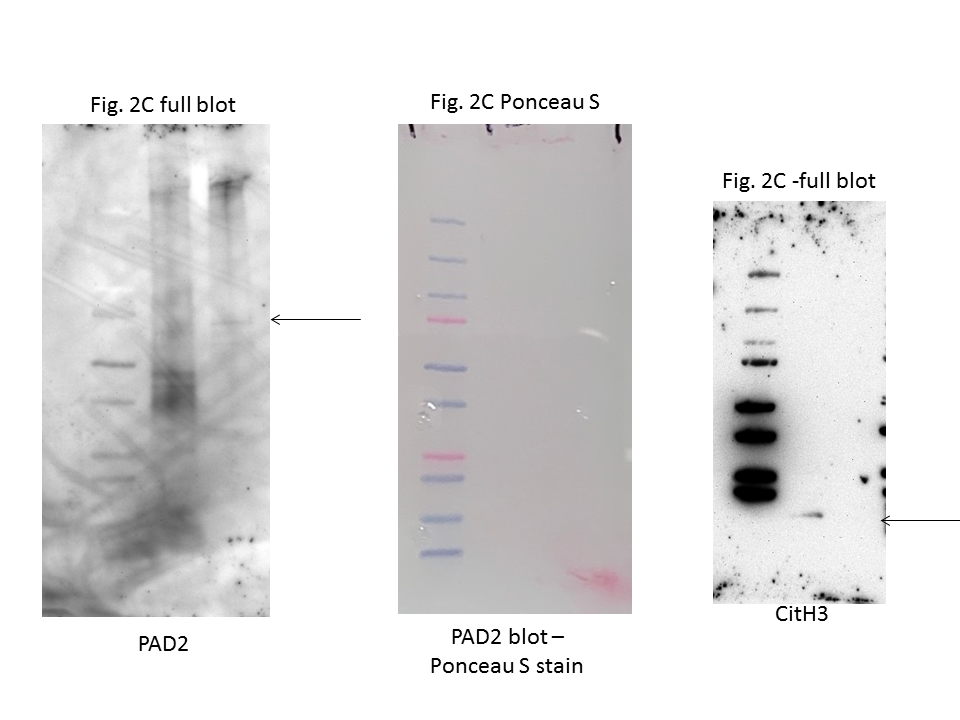

Supplement: Supplementary file 1 [file biology-10-00177-s001.zip › Appendix - Full Blots Alveolata PAD_2021.tif]
